# Neoadjuvant chemotherapy regimens in treatment of breast cancer: a systematic review and network meta-analysis protocol

**DOI:** 10.1186/s13643-018-0754-1

**Published:** 2018-06-26

**Authors:** Mona Pathak, Sada Nand Dwivedi, S. V. S. Deo, Bhaskar Thakur, Vishnubhatla Sreenivas, G. K. Rath

**Affiliations:** 1 Department of Biostatistics, All India Institute of Medical Sciences, Room No.5, New Delhi, 110029 India; 2 Department of Surgical Oncology, Dr. BRA IRCH, All India Institute of Medical Sciences, New Delhi, 110029 India; 3 Department of Radiotherapy, Dr. BRA IRCH, All India Institute of Medical Sciences, New Delhi, 110029 India

**Keywords:** Anthracycline, Taxane, Trastuzumab, Bevacizumab, Network meta-analysis, Breast cancer

## Abstract

**Background:**

Neoadjuvant chemotherapy (NACT), a standard of care for locally advanced breast cancer patients, is widely used for early breast cancer patients also. The varying role of regimens used as NACT needs to be investigated. Despite availability of some randomized controlled trials (RCTs), it is unclear which treatment regimen suits best. Further, there is no study comparing all the three regimens. Accordingly, present study will compare the efficacy of anthracyclines, taxanes, and targeted therapy administered in neoadjuvant setting on the basis of oncological outcomes and functional outcomes.

**Method/design:**

Online databases PubMed and Cochrane Register of Controlled Trials will be searched to acquire eligible studies. Further, content of relevant journals, references of relevant articles, and proceedings of major related conference will also be searched. The RCTs comparing any of abovementioned regimen as NACT on breast cancer patients will be eligible. Two reviewers independently and in duplicate will screen the records on the basis of title and abstract and complete full-text review to determine eligibility. Similarly, data extraction and risk of bias assessment will be done by two independent reviewers. The pair-wise meta-analysis as well as network meta-analysis will be conducted to assess the relative efficacy of anthracyclines, taxanes, and targeted therapy regimens.

**Discussion:**

The present systematic review will improve the understanding of the relative efficacies of the three treatment regimens and possibly guide the clinical practices by providing the current best evidence on the efficacy of various regimens of NACT in the management of breast cancer patients.

**Systematic review registration:**

PROSPERO (CRD42016027236).

**Electronic supplementary material:**

The online version of this article (10.1186/s13643-018-0754-1) contains supplementary material, which is available to authorized users.

## Background

Breast cancer, a leading cause of cancer deaths in the less developed countries with high incidence rate in developed countries, contributes more than 25% of the total number of new cases among women (World Cancer Research Fund, 2012) [1]. On the basis of severity of the disease, breast cancer is categorized in to three broad categories as Early Breast Cancer (EBC), Locally Advanced Breast Cancer (LABC) and Metastatic Breast Cancer (MBC). The treatment of breast cancer depends on the stage of disease and characteristics of the tumor. The treatment generally involves surgery, chemotherapy, radiotherapy, and hormonal therapy. Chemotherapy is given before as well as after the surgery and it targets all fast dividing cells of the body including cancer cells. Chemotherapy, given before surgery to make surgery possible, is known as neoadjuvant chemotherapy (NACT). Neoadjuvant chemotherapy is the standard of care for Locally Advanced Breast Cancer patients since its introduction in 1980s [2]. This treatment downgrades the tumor size and makes surgery possible.

The efficacy of NACT depends on the regimens used. Anthracyclines and taxanes are most active group of chemotherapy regimens used for breast cancer patients [3]. Anthracyclines involves drugs like doxorubicin and epirubicine. In the same way, taxanes have either docetaxel or paclitaxel. In addition, these drugs are generally given in combination with other drugs like fluorouracil and cyclophosphamide. Further, over past several years, new generation of cancer treatment, i.e., targeted therapy is increasingly used in the management of breast cancer patients [4]. Unlike abovementioned traditionally used regimens of chemotherapy, targeted therapy targets cancer cells but not the fast dividing normal cells. Targeted therapy includes various drugs like trastuzumab, pertuzumab, laptinib, and bevacizumab. Generally, these targeted therapy drugs are administered along with standard chemotherapy drugs.

The available evidences from randomized controlled trials are contradictory as some favored taxanes over anthracyclines [5, 6] for pathological complete response (pCR), while some showed the other way [7, 8]. In the same way, contradictory results were also reported on the efficacy of the additional use of targeted therapy like trastuzumab and bevacizumab [9–12]. Likewise, contradictory findings are also reported regarding survival outcomes while comparing various pairs of these regimens [7–9, 12–15]. In view of this, systematic review involving all these three regimens may be clinically useful. However, there is only one review which has examined the efficacy of only taxanes in comparison with anthracyclines in neoadjuvant setting [16]. Another systematic review assessed the efficacy of adding bevacizumab to anthracycline- and/or taxane-based chemotherapy [17]. Further, two more systematic reviews assessed the efficacy of adding trastuzumab to anthracycline- and/or taxane-based chemotherapy exclusively for HER2 positive patients [18, 19]. In summary, literature review did not reveal any systematic review comparing the relative efficacy of all the three viz., anthracyclines, taxanes or targeted therapy in neoadjuvant setting. Also, a known most effective treatment regimen is obvious to enhance better outcomes. Keeping in view of these points, this review comparing relative effectiveness of all three regimens among breast cancer patients is very much needed.

This study primarily aims to compare three regimens, i.e., anthracyclines, taxanes, and targeted therapy in neoadjuvant setting. The efficacy of these regimens will be assessed by pair-wise meta-analysis, and these results will also be complemented with mixed treatment comparison using network meta-analysis. As a targeted therapy, trastuzumab and bevacizumab are conventionally given to HER2-positive and HER2-negative patients respectively, the efficacy of each of these two drugs, i.e., trastuzumab and Bevacizumab will also be meta-analyzed separately.

The objectives of this study are as follows:

1. Systematic review of the RCTs comparing any pair of anthracyclines (epirubicine or doxorubicin), taxanes (Paclitaxel or docetaxel) or targeted therapies (trastuzumab, bevacizumab, laptinib, gefitinib, evirolimus, and pertuzumab) in terms of oncological and functional outcomes among breast cancer patients.
2. Meta-analysis for the following comparison depending on the availability of suitable comparable and meta-analyzable studies:

- *Pair-wise meta-analysis*

- To assess the efficacy of Taxanes in comparison with anthracyclines as NACT
- To assess the efficacy of adding targeted therapy along with NACT
- To assess the efficacy of adding trastuzumab along with NACT
- To assess the efficacy of adding bevacizumab along with NACT

- *Network meta-analysis*

3. To assess the relative efficacy of anthracyclines, taxanes, and targeted therapy in neoadjuvant setting

## Methods/design

### Design

This protocol is designed as per the guidelines of Preferred Reporting Items for Systematic Review and Meta-Analysis Protocol (PRISMA-P) [20]**.**

### Protocol registration

Our protocol has been registered on PROSPERO (CRD42016027236) [21].

### Inclusion criteria

The inclusion criteria as per PICOT are listed below:

- *Population*: Non-metastatic female breast cancer patients
- *Interventions and comparators*: As per the objectives listed earlier, the interventions and comparators are listed in the following Table 1.
- *Outcomes:* The efficacy of various regimens and their combinations will be assessed on the basis of following outcomes.
  - *Primary outcomes*: The primary outcomes are:
    i. *Pathological complete response*: pCR is defined as complete response of primary as well as axilla or of primary regardless of axilla) [22]
    ii. *Overall response*: OR is defined as complete clinical response (complete disappearance of clinically palpable tumor) or clinical partial response (more than 50% reduction in tumor volume).
    iii. *Breast conserving surgery rate*: BCS rate is defined as rate of breast conserving surgeries, i.e., lumpectomy (removal of lump only) or BCS (removal of partial breast including tumor as well as some normal tissues) among the total surgeries.
  - *Secondary outcomes*: Secondary outcomes include:
    iv. *Clinical complete response*: cCR is defined as complete disappearance of clinically palpable tumor.
    v. *Pathological complete response of primary with DCIS*: It is defined as complete response of primary regardless of axilla but allowing for ductal carcinoma in situ.
    vi. *Loco-regional recurrence*: it is defined as recurrence of carcinoma to local or regional area.
    vii. *Distant Metastasis*: It is defined as time from randomization to recurrence of carcinoma to distant area of the body.
    viii. *Disease-free survival*: It is defined as time from randomization to recurrence of carcinoma to local or distant area of the body.
    ix. *Overall survival*: It is defined as time from randomization to death from any cause.

**Table 1:** Objective-specific interventions and comparators

Objectives	Intervention	Comparator
To assess the efficacy of taxanes in comparison to anthracyclines as NACT	Taxanes	Anthracyclines
	Taxanes with anthracyclines	
To assess the efficacy of targeted therapy along with NACT	Targeted therapy with anthracyclines	Anthracyclines
	Targeted therapy with taxanes	Taxanes
	Targeted therapy with anthracyclines and taxanes	Anthracyclines and taxanes
To assess the efficacy of trastuzumab along with NACT	Trastuzumab with anthracyclines	Anthracyclines
	Trastuzumab with taxanes	Taxanes
	Trastuzumab with anthracyclines and taxanes	Anthracyclines and taxanes
To assess the efficacy of Bevacizumab along with NACT	Bevacizumab with anthracyclines	Anthracyclines
	Bevacizumab with taxanes	Taxanes
	Bevacizumab with anthracyclines and taxanes	Anthracyclines and taxanes
Network Meta-analysis	All possible and feasible pairs of regimen comparison	

NACT- Neoadjuvant Chemotherapy

### Time

Randomized controlled trials were identified by searching PubMed and Cochrane database of controlled trials on and up to 28 April 2017.

All those RCTs satisfying abovementioned criteria and published in English language will be included. Relevant studies even published in other language will also be included if their English translation is available. Corresponding authors of studies published in other languages will be contacted for possible English translation of their articles. The eligible RCTs should however measure at least one of the considered outcomes. Although, it is obvious to get advancement in the methodology over time, but it may not influence the pooled relative efficacy as we are considering only RCTs comparing the different regimens in parallel setting.

### Information sources and search

#### Bibliographic databases

The eligible RCTs will be identified using PubMed and Cochrane Central Register of Controlled Trial (CENTRAL). Further, it is worthwhile to mention here that CENTRAL database is comprised of relevant records retrieved from MEDLINE, EMBASE, Cochrane review group specialized registers and hand search. So it will be less likely to miss the relevant record after including CENTRAL. Still, the reference list of relevant article will also be searched. Abstracts of major conferences as San Antonio Breast Cancer Symposia, European Breast Cancer Conference, and Annual meeting of American Cancer Association will also be searched. All the studies included in earlier systematic reviews/meta analyses, if any, will be included.

### Search limits

At the stage of searching of online databases, it will not be restricted on the basis of language or publication time period.

### Search terms

Search strategy is developed as per Cochrane checklist for developing search strategy [23]. Text words are defined for Population (Breast Cancer), Intervention (Anthracycline, Taxane, Targeted therapy) and randomized controlled trails. Synonyms of these text words considering brand names of the drugs as well are identified. Synonyms of same text words were combined with “OR” operators. However, different text words, i.e., for Breast Cancer, Interventions and RCT were combined with “AND” operator. The detailed strategy for PubMed and Cochrane databases are given in Appendix.

### Study selection

The extracted records retrieved by both of the databases will be merged and duplicates will be removed on the basis of title and year of publication using MS Excel 2007. In the first phase, two reviewers with experience in health research methodology will screen title and available abstracts independently and in duplicate and provide the reason for non-screening of the article. For the potentially eligible articles screened by any of the reviewers, the full text will be acquired and assessed against predefined inclusion criteria. Reviewers will resolve the disagreement by consensus or, if discrepancies persist, through discussion with SVS and/or SND. After full-text review, in case of multiple records available under single study focus will be to consider the most recent publication. However, some of the relevant information included only in older publication will also be considered.

### Data collection process and data item

The data abstraction form has been designed as per the Cochrane guideline of systematic reviews of intervention (Additional file 1). The two reviewers MP and BT will abstract the data independently and in duplicate for each eligible study. SND and VS will supervise the data abstraction by MP and BT respectively. Any clinical discrepancy will be resolved by discussion with SVS or GKR.

The following information will be extracted from the eligible full-text studies:

- Publication details: year, language, country, authors, journals, phase
- Baseline factors: age, menopause status, cancer stage, hormone status (ER, PR, HER2), grade
- Inclusion criteria
- Size of study population: overall as well as in each arm
- Intervention and comparator details
- Follow-up time
- Treatment: regimen and doses, radiotherapy, hormone therapy; and duration of treatment
- Outcome variables

### Risk of bias assessment

Two reviewers engaged in data abstraction will assess risk of bias within each of the study by the Cochrane Collaboration’s tool for assessing risk of bias [23, 24]. It will be performed under the following key domains: Random sequence generation and allocation concealment for selection bias; blinding of participants and personnel (performance bias); blinding of outcome assessment (detection bias); incomplete outcome data (attrition bias); selective reporting of outcome (reporting bias) and other biases. For each of the domain, reviewers will respond as “Definitely Yes,” “Probably yes,” “Probably No,” and “Definitely No”. The former two will be assigned as low risk of bias and the later ones as high risk of bias. Any unresolved disagreement between the reviewers will be resolved by SVS, SND or VS.

### Data synthesis and analysis

The effect size under consideration will be risk ratio for pathological complete response, clinical complete response, overall response, and breast-conserving surgery. However, for overall survival, disease-free survival and time to loco-regional recurrence, being time to event outcomes, the effect size will be hazard ratio. The hazard ratio, if not reported, will be extracted from the survival curves and event rates by the method described by Parmar et al. [25].

The statistical heterogeneity will be assessed by Cochrane Q statistic and *I*^2^ statistic defined as proportion of the variance that is due to heterogeneity (true difference between study) alone rather than by chance [26, 27]. In case of very low extent of heterogeneity, i.e., (*I*^2^ = 0–25%) fixed effect method, and in case of moderate to high degree of heterogeneity (i.e., above 25%), other analytical methods like random effect method and/or weighted least square method will be used for analysis.

All analysis will be performed using Stata 14 (StataCorp, Texas, USA) and RevMan 5.3.3, Copenhagen: The Nordic Cochrane Centre, The Cochrane Collaboration, 2014. The pair-wise as well as network meta-analysis will be performed for each of the considered outcomes. RevMan will be used for systematic review, i.e., systematically collecting the study level characteristics. However, Stata will be used to perform Meta-analysis.

### Direct comparison meta-analysis and subgroup analysis

The pair-wise meta-analysis for previously listed objective will be performed using the trials having head to head comparison of the two classes of treatment regimens.

The subgroup meta-analysis will also be performed on the basis of quality of studies, i.e., for good quality studies (having low risk of bias) and studies having moderate to higher risk of bias. The subgroup meta-analysis of the trials comparing anthracycline with taxane, and anthracycline v/s anthracycline along with taxane will also be performed separately. As per the measured heterogeneity, conventional methods of meta-analysis, i.e., fixed effect method or random effect method, with inverse variance weights will be used. For survival outcomes, extracted log of hazard ratio and its standard error will be used; however, for binary outcomes, exact counts will be meta-analyzed. These methods are described briefly as below:

#### Fixed effect method

The fixed effect method assumes that each of the studies measures a single fixed effect size (μ) and variation in the effect size (if any) is due to chance alone. Statistically, it assumes that the effect size (*y*_*i*_) distribution follows normal distributed with mean (μ) and variance (*v*_*i*_), i.e., y_*i*_~*N*(*μ*, *v*_*i*_) where ${v}_i={\sigma}_i^2$. The individual study effect size can be presented as *y*_*i*_ = *μ* + *ε*_*i*_ where *ε*_*i*_ is the sampling error and ${\varepsilon}_i\sim N\left(0,{\sigma}_i^2\right)$ for *i* = 1, 2…*k*; *k* is the number of studies included in the meta-analysis. The weight *w*_*i*_ associated with *i*^th^ study is the inverse of the variance (${\sigma}_i^2$) of effect size of the considered outcome, ${w}_i=1/{\sigma}_i^2$ for that study. The pooled effect size (${\widehat{\mu}}_F$) considering these weights may be obtained as:

1 $$ {\widehat{\mu}}_F=\sum {y}_i{w}_i/\sum {w}_i $$

Further, the variance of this pooled effect size is the inverse of sum of weights:

2 $$ V\left({\widehat{\mu}}_F\right)=1/\sum {w}_i $$

#### Random effect method

Unlike fixed effect method, random effect method assumes that study level effect size is a random sample out of the infinite studies following the same inclusion criteria for meta-analysis. Random effect method considers that study level effect size may vary systematically because of change in its population, intervention, comparator, outcome definition, and used design. In other words, the deviation (*θ*_*i*_) between individual-level study effect size (*y*_*i*_) and the true effect (*μ*) in the population exceeds that due to sampling variation (*ε*_*i*_) alone. The individual study effect size can be modeled as:

3 $$ {y}_i=\mu +{\theta}_i+{\varepsilon}_i $$

Further, *θ*_*i*_ depend on between study variance (*τ*^2^) while *ε*_*i*_ depends on the within study variance (${\sigma}_i^2$) alone and these entities distributed normally as *θ*_*i*_~*N*(0, *τ*^2^) and ${\varepsilon}_i\sim N\left(0,{\sigma}_i^2\right)$ respectively. Since random effect method considers two source of variation as between study variance as well as within study variance, the weights (*w*′_*i*_) associated with individual studies are inverse of this total variation:

4 $$ w{\hbox{'}}_i=1/\left({\sigma}_i^2+{\tau}^2\right) $$

The pooled effect estimate of *y*_*i*_ may be given as below:

5 $$ {\widehat{\mu}}_R=\sum {y}_iw{\hbox{'}}_i/\sum w{\hbox{'}}_i $$

where *y*_*i*_ is distributed as ${y}_i\sim N\left(\mu, {\sigma}_i^2+{\tau}^2\right).$

Further, the variance of this estimated effect size may be represented as:

6 $$ V\left({\widehat{\mu}}_R\right)=1/\sum w{\hbox{'}}_i $$

#### Network meta-analysis

The assumptions for network meta-analysis, i.e., ‘*consistency*’ for mixed treatment comparison and ‘*similarity*’ for indirect comparison, will be examined before analysis. The inconsistency (i.e., disparity between direct and indirect estimates) will be tested by mixed comparison modeling using inconsistency factor for each closed loop [28]. The similarity assumption will be qualitatively examined by visualizing the distribution of potential effect modifiers across the RCTs. The network meta-analysis model as a multivariate meta-regression of the effect size associated with regimens [29] will be developed as follows:

Let *T* treatments were compared in *n* studies and *Ti* represents the number of treatment compared in *i*^th^ study. A total of ^*Ti*^C_2_ contrasts could be estimated from these treatment comparisons. But, out of these only *T*_*i*_ − 1 contrasts are orthogonal, rest of the contrasts can be estimated as linear combinations of these orthogonal contrasts. The vector **y** of estimated contrasts across all the studies may be expressed as:

7 $$ \boldsymbol{y}=\left\{{y}_{1,1},\dots ..{y}_{1,T-1},{y}_{2,1},\dots \dots .{y}_{2,T-1},{y}_{n,1},\dots \dots .{y}_{n,T-1}\right\} $$

where *y*_*ij*_ represent *j*^th^ contrast from *i*^th^ study

The length of vector y is Σ(*T*_*i*_ − 1).

In case of consistency, the random effect model to estimate **y** is given by

8 $$ \boldsymbol{y}=X\boldsymbol{\mu} +\boldsymbol{\beta} +\boldsymbol{\epsilon} $$

where **μ** is a vector of independent contrasts, **β** is a vector of random effects taking care of between study heterogeneity, and **ε** expresses the vector of random error in **y**, i.e., within study heterogeneity. **β** and **ε** follow normal distribution with mean zero and respective variance covariance matrix Ω and V. X is a design matrix describing the independent and dependent contrast being estimated from **y**. However, in case of inconsistency, the model described in Eq. 8 will be:

9 $$ \boldsymbol{y}=X\boldsymbol{\mu} +\boldsymbol{\beta} +Z\boldsymbol{\omega} +\boldsymbol{\epsilon} $$

Here, ω is between design heterogeneity. It is a vector of length *G* which is less than or equal to the total number of contrasts. *G* is the total number of different comparisons being made in the network. The extracted covariates, significant as per meta-regression, will also be adjusted in the model by adding in the equation.

The treatment effect will be reported on the basis of 95% confidence intervals. The possible ranking of treatment, if meaningful, will be done by surface below the cumulative step function for each regimen [30]. The probabilities of each regimen found to be best, second best, and likewise in comparison with a common control, i.e., anthracycline alone, will be calculated [30]. The strength of evidence and credibility of our study will be provided using critically apprised guide for network meta-analysis [31].

### Anticipated structure of network

The nodes of network will represent treatment regimens as (1) anthracycline, (2) taxane, (3) anthracycline with taxane, (4) anthracycline + trastuzumab, (5) anthracycline + bevacizumab, (6) taxane + trastuzumab, (7) taxane + bevacizumab, (8) anthracycline + taxane+ trastuzumab, and (9) anthracycline + taxane + bevacizumab, subject to the condition they are compared in eligible RCTs. The connected lines of the network will represent the number of RCTs comparing such two treatment regimens.

### Confidence in pooled estimate of effect

Two independent reviewers will assess the confidence of pooled treatment effect using GRADE (Grading of Recommendation, Assessment, development and Evaluation) rating system [32]. GRADE rating system involves five categories of limitation of RCTs as (1) Risk of Bias, (2) Consistency, (3) Directness, (4) Impression and (5) Reporting Bias. On the basis of these five categories, the confidence will be graded as high, moderate, low, and very low [32]. The publication bias will be assessed by funnel plot and Egger’s test. However, in case of network meta-analysis, each treatment comparison has its own summary effect, so each comparison naturally has a line of symmetry. As evident from it, all treatment comparisons involved in the network do not have single reference line of symmetry. Accordingly, comparison adjusted funnel plots will be used in network meta-analysis. In case any small RCTs involving extreme treatment effect (publication bias)/design issues/risk of bias, the sensitivity analysis will be performed by including and excluding such RCTs to assess the robustness of evidence. The result of pair-wise meta-analysis will be reported along with network meta-analysis to make it clear how comparable are the network meta-analysis results with pair-wise analysis. The results of only network meta-analysis will be reported for the treatment comparison, when no head to head comparison available.

### Dissemination of work

The results of the present study will be widely disseminated among end users including oncologists, policy makers, and researchers working in the similar area through presentation in national and international conferences, symposiums, and meetings.

## Discussion

The present systematic review will evaluate the relative efficacy of various regimens used in neo-adjuvant setting. Further, it will also review the quality of evidence using GRADE approach [32]. To the best of our knowledge, it will be the first study comparing the relative efficacy of anthracycline- and taxane-based chemotherapy and targeted therapy regimens along with NACT in the management of breast cancer patients. Accordingly, it will facilitate evidence-based management of breast cancer patients. Hence, this systematic review will be beneficial for wide audience including breast cancer patients, oncology professionals, insurers, policy makers, and researchers working in the field of oncology.

### Additional file

Additional file 1: Data extraction form designed to extract trial level data. (DOC 494 kb)

## Appendix

### Medline Search Strategy

((breast and AND (cancer OR tumour OR tumor OR neoplas*)) OR (“Breast Neoplasms”[Mesh])) AND (“Taxoids”[Mesh] OR docetaxel OR paclitaxel OR taxol OR taxotere OR abraxane OR nab-paclitaxel OR taxan* OR trastuzumab OR herceptin OR Everolimus OR lapatinib OR pertuzumab OR bevacizumab OR palbociclib OR Doxorubicin OR Adriamycin OR epirubicin OR Idarubicin OR anthracycline) AND (Neoadjuvant OR preoperat* OR upfront OR Primary) AND (“Drug Therapy”[Mesh] OR Chemotherapy) AND Randomized Controlled Trial[ptyp]

### CENTRAL Search Strategy

Search strategy Cochrane Central Register of Controlled trials is given in Table 2.

**Table 2:** CENTRAL Search strategy

#01	MeSH descriptor: [Breast Neoplasms] explode all trees
#02	breast and (cancer or tumour or tumor or neoplas*)
#03	#1 or #2
#04	neoadjuvant or preoperat* or upfront or primary
#05	therapy or chemotherapy
#06	MeSH descriptor: [Drug Therapy] explode all trees
#07	#5 or #6
#08	MeSH descriptor: [Taxoids] explode all trees
#09	docetaxel or paclitaxel or taxol or taxotere or abraxane or nab-paclitaxel or taxan*
#10	#8 or #9
#11	trastuzumab or herceptin or Everolimus or lapatinib or pertuzumab or bevacizumab or palbociclib
#12	Doxorubicin or Adriamycin or epirubicin or Idarubicin or anthracycline
#13	#10 or #11 or #12
#14	#4 near #7
#15	#3 and #13 and #14 in Trials

## Abbreviations

BCS: Breast Conserving Surgery
cCR: Clinical Complete Response
cPR: Clinical Partial Response
DFS: Disease-free survival
DR: Distal recurrence
LRR: Loco-regional recurrence
NACT: Neoadjuvant chemotherapy
OR: Overall Response
OS: Overall survival
pCR: Pathological complete response
PD: Progressive disease
SD: Stable disease
