# Supplementary material for: Neoadjuvant chemotherapy regimens in treatment of breast cancer: a systematic review and network meta-analysis protocol
Source: Syst Rev. 2018 Jun 26;7:89. doi: 10.1186/s13643-018-0754-1 (PMC6020442; doi:10.1186/s13643-018-0754-1)
Supplement: Supplementary file 1 — Data extraction form designed to extract trial level data. (DOC 494 kb) [file 13643_2018_754_MOESM1_ESM.doc]

Data collection form for intervention reviews for RCT Comparing regimens of NACT based on the Cochrane guidelines

| Review title or ID |  |
| --- | --- |
| Study ID *(surname of first author and year first full report of study was published e.g. Smith 2001)* |  |
| Report ID |  |
| Report ID of other reports of this study including errata or retractions |  |
| Notes | |

# General Information

| Date form completed *(dd/mm/yyyy)* |  |
| --- | --- |
| Name/ID of person extracting data |  |
| Reference citation |  |
| Study author contact details |  |
| Publication type *(e.g. full report, abstract, letter)* |  |
| Notes: | |

# Study eligibility

| Study Characteristics | Eligibility criteria  *(Insert inclusion criteria for each characteristic as defined in the Protocol)* | | Eligibility criteria met? | | | Location in text or source *(pg & ¶/fig/table/other)* |
| --- | --- | --- | --- | --- | --- | --- |
|  |  |  | Yes | No | Unclear |  |
| Type of study | Randomised controlled trial | |  |  |  |  |
|  | Quasi-randomised controlled trial | |  |  |  |  |
| Participants | Breast Cancer Patients | |  |  |  |  |
| Types of intervention | Anthracycline, Taxane, Trastuzumab based interventions | |  |  |  |  |
| Types of comparison | Anthracycline, Taxane, Trastuzumab based interventions | |  |  |  |  |
| Types of outcome measures | Pathological complete response, overall survival, relapse free survival, time to loco-regional recurrence, time to distal recurrence, breast conserving surgery, toxicity | |  |  |  |  |
| INCLUDE | | EXCLUDE | | | | |
| Reason for exclusion |  | | | | | |
| Notes: | | | | | | |

**DO NOT PROCEED IF STUDY EXCLUDED FROM REVIEW**

# Characteristics of included studies

## Methods

|  | **Descriptions as stated in report/paper** | | **Location in text or source** *(pg & ¶/fig/table/other)* |
| --- | --- | --- | --- |
| **Aim of study** *(e.g. efficacy, equivalence, pragmatic)* |  | |  |
| **Design** *(e.g. parallel, crossover, non-RCT)* |  | |  |
| **Unit of allocation** |  | |  |
| **Start date** |  | |  |
| **End date** |  | |  |
| **Duration of participation** *(from recruitment to last follow-up)* |  | |  |
| **Ethical approval needed/ obtained for study** | Yes No Unclear |  |  |
| **Notes:** | | | |

## Participants

|  | Description  *Include comparative information for each intervention or comparison group if available* | Location in text or source *(pg & ¶/fig/table/other)* |
| --- | --- | --- |
|  | Overall |  |
| Population description *(from which study participants are drawn)* |  |  |
| Setting *(including location and social context)* |  |  |
| Inclusion criteria |  |  |
| Exclusion criteria |  |  |
| Method of recruitment of participants *(e.g. phone, mail, clinic patients)* |  |  |
| Informed consent obtained | Yes No Unclear |  |
| Total no. randomised *(or total pop. at start of study for non-RCTs)* |  |  |
| Baseline imbalances |  |  |
| Withdrawals and exclusions *(if not provided below by outcome)* |  |  |
| Baseline factors Overall and arm wise | | |
| Age |  |  |
| Race/ethnicity |  |  |
| Menopause |  |  |
| Clinical Stage |  |  |
| Pathological Stage |  |  |
| Tumour size |  |  |
| Tumour Grade |  |  |
| Nodal Status |  |  |
| Metastatic |  |  |
| Lymph node involvement |  |  |
| ER Status Positive |  |  |
| PR Status Positive |  |  |
| HER2 Status Positive |  |  |
| TNBC |  |  |
| Co-morbidities |  |  |
| Other relevant socio demographics |  |  |
| Subgroups measured |  |  |
| Subgroups reported |  |  |
| Notes: | | |

## Intervention groups

*Copy and paste table for each intervention and comparison group*

Intervention Group 1

|  | Description as stated in report/paper | Location in text or source *(pg & ¶/fig/table/other)* |
| --- | --- | --- |
| Group name |  |  |
| No. randomised to group *(specify whether no. people or clusters)* |  |  |
| Theoretical basis *(include key references)* |  |  |
| Description *(include sufficient detail for replication, e.g. content, dose, components)* |  |  |
| Duration of treatment period |  |  |
| Regimen in detail |  |  |
| Timing *(e.g. frequency, duration of each episode)* |  |  |
| Delivery *(e.g. mechanism, medium, intensity, fidelity)* |  |  |
| Providers *(e.g. no., profession, training, ethnicity etc. if relevant)* |  |  |
| Co-interventions |  |  |
| Economic information *(i.e. intervention cost, changes in other costs as result of intervention)* |  |  |
| Resource requirements *(e.g. staff numbers, cold chain, equipment)* |  |  |
| Integrity of delivery |  |  |
| Compliance |  |  |
| Notes: | | |

**Intervention Group 2**

|  | Description as stated in report/paper | Location in text or source *(pg & ¶/fig/table/other)* |
| --- | --- | --- |
| Group name |  |  |
| No. randomised to group *(specify whether no. people or clusters)* |  |  |
| Theoretical basis *(include key references)* |  |  |
| Description *(include sufficient detail for replication, e.g. content, dose, components)* |  |  |
| Duration of treatment period |  |  |
| Timing *(e.g. frequency, duration of each episode)* |  |  |
| Delivery *(e.g. mechanism, medium, intensity, fidelity)* |  |  |
| Providers *(e.g. no., profession, training, ethnicity etc. if relevant)* |  |  |
| Co-interventions |  |  |
| Economic information *(i.e. intervention cost, changes in other costs as result of intervention)* |  |  |
| Resource requirements *(e.g. staff numbers, cold chain, equipment)* |  |  |
| Integrity of delivery |  |  |
| Compliance |  |  |
| Notes: | | |

**Intervention Group 3 if available**

|  | Description as stated in report/paper | Location in text or source *(pg & ¶/fig/table/other)* |
| --- | --- | --- |
| Group name |  |  |
| No. randomised to group *(specify whether no. people or clusters)* |  |  |
| Theoretical basis *(include key references)* |  |  |
| Description *(include sufficient detail for replication, e.g. content, dose, components)* |  |  |
| Duration of treatment period |  |  |
| Timing *(e.g. frequency, duration of each episode)* |  |  |
| Delivery *(e.g. mechanism, medium, intensity, fidelity)* |  |  |
| Providers *(e.g. no., profession, training, ethnicity etc. if relevant)* |  |  |
| Co-interventions |  |  |
| Economic information *(i.e. intervention cost, changes in other costs as result of intervention)* |  |  |
| Resource requirements *(e.g. staff numbers, cold chain, equipment)* |  |  |
| Integrity of delivery |  |  |
| Compliance |  |  |
| Notes: | | |

## Outcomes

*Copy and paste table for each outcome.*

**Outcome 1 (Overall Survival)**

|  | Description as stated in report/paper | | Location in text or source *(pg & ¶/fig/table/other)* |
| --- | --- | --- | --- |
| Outcome name |  | |  |
| Time points measured *(specify whether from start or end of intervention)* |  | |  |
| Time points reported |  | |  |
| Outcome definition *(with diagnostic criteria if relevant)* |  | |  |
| Person measuring/ reporting |  | |  |
| Unit of measurement *(if relevant)* |  | |  |
| Scales: upper and lower limits *(indicate whether high or low score is good)* |  | |  |
| Is outcome/tool validated? | Yes No Unclear |  |  |
| Imputation of missing data *(e.g. assumptions made for ITT analysis)* |  | |  |
| Assumed risk estimate *(e.g. baseline or population risk noted in Background)* |  | |  |
| Power *(e.g. power & sample size calculation, level of power achieved)* |  | |  |
| Notes: | | | |

**Outcome 2 (Disease free Survival)**

|  | Description as stated in report/paper | | Location in text or source *(pg & ¶/fig/table/other)* |
| --- | --- | --- | --- |
| Outcome name |  | |  |
| Time points measured *(specify whether from start or end of intervention)* |  | |  |
| Time points reported |  | |  |
| Outcome definition *(with diagnostic criteria if relevant)* |  | |  |
| Person measuring/ reporting |  | |  |
| Unit of measurement *(if relevant)* |  | |  |
| Scales: upper and lower limits *(indicate whether high or low score is good)* |  | |  |
| Is outcome/tool validated? | Yes No Unclear |  |  |
| Imputation of missing data *(e.g. assumptions made for ITT analysis)* |  | |  |
| Assumed risk estimate *(e.g. baseline or population risk noted in Background)* |  | |  |
| Power *(e.g. power & sample size calculation, level of power achieved)* |  | |  |
| Notes: | | | |

**Outcome 3 (time to loco-regional recurrence)**

|  | Description as stated in report/paper | | Location in text or source *(pg & ¶/fig/table/other)* |
| --- | --- | --- | --- |
| Outcome name |  | |  |
| Time points measured *(specify whether from start or end of intervention)* |  | |  |
| Time points reported |  | |  |
| Outcome definition *(with diagnostic criteria if relevant)* |  | |  |
| Person measuring/ reporting |  | |  |
| Unit of measurement *(if relevant)* |  | |  |
| Scales: upper and lower limits *(indicate whether high or low score is good)* |  | |  |
| Is outcome/tool validated? | Yes No Unclear |  |  |
| Imputation of missing data *(e.g. assumptions made for ITT analysis)* |  | |  |
| Assumed risk estimate *(e.g. baseline or population risk noted in Background)* |  | |  |
| Power *(e.g. power & sample size calculation, level of power achieved)* |  | |  |
| Notes: | | | |

**Outcome 4 (Time to Distal recurrence)**

|  | Description as stated in report/paper | | Location in text or source *(pg & ¶/fig/table/other)* |
| --- | --- | --- | --- |
| Outcome name |  | |  |
| Time points measured *(specify whether from start or end of intervention)* |  | |  |
| Time points reported |  | |  |
| Outcome definition *(with diagnostic criteria if relevant)* |  | |  |
| Person measuring/ reporting |  | |  |
| Unit of measurement *(if relevant)* |  | |  |
| Scales: upper and lower limits *(indicate whether high or low score is good)* |  | |  |
| Is outcome/tool validated? | Yes No Unclear |  |  |
| Imputation of missing data *(e.g. assumptions made for ITT analysis)* |  | |  |
| Assumed risk estimate *(e.g. baseline or population risk noted in Background)* |  | |  |
| Power *(e.g. power & sample size calculation, level of power achieved)* |  | |  |
| Notes: | | | |

**Outcome 5 (Breast Conserving Surgery)**

|  | Description as stated in report/paper | | Location in text or source *(pg & ¶/fig/table/other)* |
| --- | --- | --- | --- |
| Outcome name |  | |  |
| Time points measured *(specify whether from start or end of intervention)* |  | |  |
| Time points reported |  | |  |
| Outcome definition *(with diagnostic criteria if relevant)* |  | |  |
| Person measuring/ reporting |  | |  |
| Unit of measurement *(if relevant)* |  | |  |
| Scales: upper and lower limits *(indicate whether high or low score is good)* |  | |  |
| Is outcome/tool validated? | Yes No Unclear |  |  |
| Imputation of missing data *(e.g. assumptions made for ITT analysis)* |  | |  |
| Assumed risk estimate *(e.g. baseline or population risk noted in Background)* |  | |  |
| Power *(e.g. power & sample size calculation, level of power achieved)* |  | |  |
| Notes: | | | |

**Outcome 6 (pathological complete response)**

|  | Description as stated in report/paper | | Location in text or source *(pg & ¶/fig/table/other)* |
| --- | --- | --- | --- |
| Outcome name |  | |  |
| Time points measured *(specify whether from start or end of intervention)* |  | |  |
| Time points reported |  | |  |
| Outcome definition *(with diagnostic criteria if relevant)* |  | |  |
| Person measuring/ reporting |  | |  |
| Unit of measurement *(if relevant)* |  | |  |
| Scales: upper and lower limits *(indicate whether high or low score is good)* |  | |  |
| Is outcome/tool validated? | Yes No Unclear |  |  |
| Imputation of missing data *(e.g. assumptions made for ITT analysis)* |  | |  |
| Assumed risk estimate *(e.g. baseline or population risk noted in Background)* |  | |  |
| Power *(e.g. power & sample size calculation, level of power achieved)* |  | |  |
| Notes: | | | |

**Outcome 7 (Clinical Response)**

|  | Description as stated in report/paper | | Location in text or source *(pg & ¶/fig/table/other)* |
| --- | --- | --- | --- |
| Outcome name |  | |  |
| Time points measured *(specify whether from start or end of intervention)* |  | |  |
| Time points reported |  | |  |
| Outcome definition *(with diagnostic criteria if relevant)* |  | |  |
| Person measuring/ reporting |  | |  |
| Unit of measurement *(if relevant)* |  | |  |
| Scales: upper and lower limits *(indicate whether high or low score is good)* |  | |  |
| Is outcome/tool validated? | Yes No Unclear |  |  |
| Imputation of missing data *(e.g. assumptions made for ITT analysis)* |  | |  |
| Assumed risk estimate *(e.g. baseline or population risk noted in Background)* |  | |  |
| Power *(e.g. power & sample size calculation, level of power achieved)* |  | |  |
| Notes: | | | |

**Outcome 8 (Treatment toxicity)**

|  | Description as stated in report/paper | | Location in text or source *(pg & ¶/fig/table/other)* |
| --- | --- | --- | --- |
| Outcome name |  | |  |
| Name of Toxicity measured |  | |  |
| Time points measured *(specify whether from start or end of intervention)* |  | |  |
| Time points reported |  | |  |
| Outcome definition *(with diagnostic criteria if relevant)* |  | |  |
| Person measuring/ reporting |  | |  |
| Unit of measurement *(if relevant)* |  | |  |
| Scales: upper and lower limits *(indicate whether high or low score is good)* |  | |  |
| Is outcome/tool validated? | Yes No Unclear |  |  |
| Imputation of missing data *(e.g. assumptions made for ITT analysis)* |  | |  |
| Assumed risk estimate *(e.g. baseline or population risk noted in Background)* |  | |  |
| Power *(e.g. power & sample size calculation, level of power achieved)* |  | |  |
| Notes: | | | |

**Other Outcome if reported**

|  | Description as stated in report/paper | | Location in text or source *(pg & ¶/fig/table/other)* |
| --- | --- | --- | --- |
| Outcome name |  | |  |
| Time points measured *(specify whether from start or end of intervention)* |  | |  |
| Time points reported |  | |  |
| Outcome definition *(with diagnostic criteria if relevant)* |  | |  |
| Person measuring/ reporting |  | |  |
| Unit of measurement *(if relevant)* |  | |  |
| Scales: upper and lower limits *(indicate whether high or low score is good)* |  | |  |
| Is outcome/tool validated? | Yes No Unclear |  |  |
| Imputation of missing data *(e.g. assumptions made for ITT analysis)* |  | |  |
| Assumed risk estimate *(e.g. baseline or population risk noted in Background)* |  | |  |
| Power *(e.g. power & sample size calculation, level of power achieved)* |  | |  |
| Notes: | | | |

## Other

| Study funding sources *(including role of funders)* |  |  |
| --- | --- | --- |
| Possible conflicts of interest *(for study authors)* |  |  |
| Notes: | | |

# Risk of Bias assessment

***(See [Handbook Chapter 8](http://handbook.cochrane.org/index.htm#chapter_8/8_assessing_risk_of_bias_in_included_studies.htm). Additional domains may be added for non-randomised studies.)***

| Domain | Risk of bias | | | Support for judgement  *(include direct quotes where available with explanatory comments)* | Location in text or source *(pg & ¶/fig/table/other)* |
| --- | --- | --- | --- | --- | --- |
|  | Low | High | Unclear |  |  |
| Random sequence generation *(selection bias)* |  |  |  |  |  |
| Allocation concealment *(selection bias)* |  |  |  |  |  |
| Blinding of participants and personnel *(performance bias)* |  |  |  | Outcome group: All/ |  |
| *(if separate judgement by outcome(s) required)* |  |  |  | Outcome group: |  |
| Blinding of outcome assessment *(detection bias)* |  |  |  | Outcome group: All/ |  |
| *(if separate judgement by outcome(s) required)* |  |  |  | Outcome group: |  |
| Incomplete outcome data *(attrition bias)* |  |  |  | Outcome group: All/ |  |
| *(if separate judgement by outcome(s) required)* |  |  |  | Outcome group: |  |
| Selective outcome reporting? *(reporting bias)* |  |  |  |  |  |
| Other bias |  |  |  |  |  |
| Notes: | | | | | |

# Data and analysis

*Copy and paste the appropriate table for each outcome, including additional tables for each time point and subgroup as required.*

***Outcome 1 (Overall Survival)***

|  | | Description as stated in report/paper | | | | | Location in text or source *(pg & ¶/fig/table/other)* | |
| --- | --- | --- | --- | --- | --- | --- | --- | --- |
| Comparison Regimens | |  | | | | |  | |
| Outcome | |  | | | | |  | |
| Subgroup | |  | | | | |  | |
| Time point *(specify from start or end of intervention)* | |  | | | | |  | |
| Post-intervention or change from baseline? | |  | | | | |  | |
| Comparison 1: | | Reference Regimen: | | | | | | |
| Results if HR reported | HR | SD *(or other variance, specify)* | | | No. participants | |  | |
|  |  |  |  |  |  |  |  |  |
|  |  |  |  |  |  |  |  |  |
|  | UCI | LCI | | |  | |  |  |
|  |  |  |  |  |  |  |  |  |
|  | Cox p-value for intervention: | | | | | |  | |
| Results if no. of events are reported | Intervention | | | Comparison | | |  | |
|  | Observed no of death | | No. participants | Observed no of death | | No. participants |  |  |
|  |  |  |  |  |  |  |  |  |
| Results if not reported | Are Kaplan-meire survival curve are reported:  Yes  No | | | | | |  | |
| Comparison 2 | | Reference Regimen: | | | | | | |
| Results if HR reported | HR | SD *(or other variance, specify)* | | | No. participants | |  | |
|  |  |  |  |  |  |  |  |  |
|  |  |  |  |  |  |  |  |  |
|  | UCI | LCI | | |  | |  |  |
|  |  |  |  |  |  |  |  |  |
|  | Cox p-value for intervention: | | | | | |  | |
| Results if no. of events are reported | Intervention | | | Comparison | | |  | |
|  | Observed no of death | | No. participants | Observed no of death | | No. participants |  |  |
|  |  |  |  |  |  |  |  |  |
| Results if not reported | Are Kaplan-meire survival curve are reported:  Yes  No | | | | | |  | |
| No. missing participants | |  | |  | | |  |  |
| Reasons missing | |  | |  | | |  |  |
| No. participants moved from other group | |  | |  | | |  |  |
| Reasons moved | | Yes No Unclear | |  | | |  |  |
| Unit of analysis *(individuals, cluster/ groups or body parts)* | | Yes No Unclear | | | | |  | |
| Statistical methods used and appropriateness of these *(e.g. adjustment for correlation)* | |  | | | | |  | |
| Reanalysis required? *(specify)* | |  | |  | | |  | |
| Reanalysis possible? | |  | |  | | |  | |
| Reanalysed results | |  | | | | |  | |
|  | | | | | | | | |

***Outcome 2 (Disease free Survival)***

|  | | Description as stated in report/paper | | | | | Location in text or source *(pg & ¶/fig/table/other)* |
| --- | --- | --- | --- | --- | --- | --- | --- |
| Comparison Regimens | |  | | | | |  |
| Outcome | |  | | | | |  |
| Subgroup | |  | | | | |  |
| Time point *(specify from start or end of intervention)* | |  | | | | |  |
| Post-intervention or change from baseline? | |  | | | | |  |
| Comparison 1: | | Reference Regimen: | | | | | |
| Results if HR reported | HR | SD *(or other variance, specify)* | | | No. participants | |  |
|  |  |  |  |  |  |  |  |
|  |  |  |  |  |  |  |  |
|  | UCI | LCI | | |  | |  |
|  |  |  |  |  |  |  |  |
|  | Cox p-value for intervention: | | | | | |  |
| Results if no. of events are reported | Intervention (_________________) | | | Comparison(_________________) | | |  |
|  | Observed no of recurrence | | No. participants | Observed no of recurrence | | No. participants |  |
|  |  |  |  |  |  |  |  |
| Results if not reported | Are Kaplan-meire survival curve are reported:  Yes  No | | | | | |  |
| Comparison 2 | | Reference Regimen: | | | | | |
| Results if HR reported | HR | SD *(or other variance, specify)* | | | No. participants | |  |
|  |  |  |  |  |  |  |  |
|  |  |  |  |  |  |  |  |
|  | UCI | LCI | | |  | |  |
|  |  |  |  |  |  |  |  |
|  | Cox p-value for intervention: | | | | | |  |
| Results if no. of events are reported | Intervention (_________________) | | | Comparison(_________________) | | |  |
|  | Observed no of recurrence | | No. participants | Observed no of recurrence | | No. participants |  |
|  |  |  |  |  |  |  |  |
| Results if not reported | Are Kaplan-meire survival curve are reported:  Yes  No | | | | | |  |
| No. missing participants | |  | |  | | |  |
| Reasons missing | |  | |  | | |  |
| No. participants moved from other group | |  | |  | | |  |
| Reasons moved | | Yes No Unclear | |  | | |  |
| Unit of analysis *(individuals, cluster/ groups or body parts)* | | Yes No Unclear | | | | |  |
| Statistical methods used and appropriateness of these *(e.g. adjustment for correlation)* | |  | | | | |  |
| Reanalysis required? *(specify)* | |  | |  | | |  |
| Reanalysis possible? | |  | |  | | |  |
| Reanalysed results | |  | | | | |  |
|  | | | | | | | |

***Outcome 3 (time to loco-regional recurrence)***

|  | | Description as stated in report/paper | | | | | Location in text or source *(pg & ¶/fig/table/other)* |
| --- | --- | --- | --- | --- | --- | --- | --- |
| Comparison Regimens | |  | | | | |  |
| Outcome | |  | | | | |  |
| Subgroup | |  | | | | |  |
| Time point *(specify from start or end of intervention)* | |  | | | | |  |
| Post-intervention or change from baseline? | |  | | | | |  |
| Comparison 1: | | Reference Regimen: | | | | | |
| Results if HR reported | HR | SD *(or other variance, specify)* | | | No. participants | |  |
|  |  |  |  |  |  |  |  |
|  |  |  |  |  |  |  |  |
|  | UCI | LCI | | |  | |  |
|  |  |  |  |  |  |  |  |
|  | Cox p-value for intervention: | | | | | |  |
| Results if no. of events are reported | Intervention (_________________) | | | Comparison(_________________) | | |  |
|  | Observed no of local recurrence | | No. participants | Observed no of local recurrence | | No. participants |  |
|  |  |  |  |  |  |  |  |
| Results if not reported | Are Kaplan-meire survival curve are reported:  Yes  No | | | | | |  |
| Comparison 2 | | Reference Regimen: | | | | | |
| Results if HR reported | HR | SD *(or other variance, specify)* | | | No. participants | |  |
|  |  |  |  |  |  |  |  |
|  |  |  |  |  |  |  |  |
|  | UCI | LCI | | |  | |  |
|  |  |  |  |  |  |  |  |
|  | Cox p-value for intervention: | | | | | |  |
| Results if no. of events are reported | Intervention (_________________) | | | Comparison(_________________) | | |  |
|  | Observed no of recurrence | | No. participants | Observed no of recurrence | | No. participants |  |
|  |  |  |  |  |  |  |  |
| Results if not reported | Are Kaplan-meire survival curve are reported:  Yes  No | | | | | |  |
| No. missing participants | |  | |  | | |  |
| Reasons missing | |  | |  | | |  |
| No. participants moved from other group | |  | |  | | |  |
| Reasons moved | | Yes No Unclear | |  | | |  |
| Unit of analysis *(individuals, cluster/ groups or body parts)* | | Yes No Unclear | | | | |  |
| Statistical methods used and appropriateness of these *(e.g. adjustment for correlation)* | |  | | | | |  |
| Reanalysis required? *(specify)* | |  | |  | | |  |
| Reanalysis possible? | |  | |  | | |  |
| Reanalysed results | |  | | | | |  |
|  | | | | | | | |

Outcome 4: Time to Distal recurrence

|  | | Description as stated in report/paper | | | | | Location in text or source *(pg & ¶/fig/table/other)* |
| --- | --- | --- | --- | --- | --- | --- | --- |
| Comparison Regimens | |  | | | | |  |
| Outcome | |  | | | | |  |
| Subgroup | |  | | | | |  |
| Time point *(specify from start or end of intervention)* | |  | | | | |  |
| Post-intervention or change from baseline? | |  | | | | |  |
| Comparison 1: | | Reference Regimen: | | | | | |
| Results if HR reported | HR | SD *(or other variance, specify)* | | | No. participants | |  |
|  |  |  |  |  |  |  |  |
|  |  |  |  |  |  |  |  |
|  | UCI | LCI | | |  | |  |
|  |  |  |  |  |  |  |  |
|  | Cox p-value for intervention: | | | | | |  |
| Results if no. of events are reported | Intervention (_________________) | | | Comparison(_________________) | | |  |
|  | Observed no of distal recurrence | | No. participants | Observed no of distal recurrence | | No. participants |  |
|  |  |  |  |  |  |  |  |
| Results if not reported | Are Kaplan-meire survival curve are reported:  Yes  No | | | | | |  |
| Comparison 2 | | Reference Regimen: | | | | | |
| Results if HR reported | HR | SD *(or other variance, specify)* | | | No. participants | |  |
|  |  |  |  |  |  |  |  |
|  |  |  |  |  |  |  |  |
|  | UCI | LCI | | |  | |  |
|  |  |  |  |  |  |  |  |
|  | Cox p-value for intervention: | | | | | |  |
| Results if no. of events are reported | Intervention (_________________) | | | Comparison(_________________) | | |  |
|  | Observed no of distal recurrence | | No. participants | Observed no of distal recurrence | | No. participants |  |
|  |  |  |  |  |  |  |  |
| Results if not reported | Are Kaplan-meire survival curve are reported:  Yes  No | | | | | |  |
| No. missing participants | |  | |  | | |  |
| Reasons missing | |  | |  | | |  |
| No. participants moved from other group | |  | |  | | |  |
| Reasons moved | | Yes No Unclear | |  | | |  |
| Unit of analysis *(individuals, cluster/ groups or body parts)* | | Yes No Unclear | | | | |  |
| Statistical methods used and appropriateness of these *(e.g. adjustment for correlation)* | |  | | | | |  |
| Reanalysis required? *(specify)* | |  | |  | | |  |
| Reanalysis possible? | |  | |  | | |  |
| Reanalysed results | |  | | | | |  |
|  | | | | | | | |

***Outcome 5*** (Breast Conserving Surgery)

|  | Description as stated in report/paper | | | | | Location in text or source *(pg & ¶/fig/table/other)* |
| --- | --- | --- | --- | --- | --- | --- |
| Comparison |  | | | | |  |
| Outcome |  | | | | |  |
| Subgroup |  | | | | |  |
| Time point *(specify from start or end of intervention)* |  | | | | |  |
| Comparison 1 Results | Intervention (____________) | | | Comparison (_____________) | |  |
|  | No. with event | Total in group | | No. with event | Total in group |  |
|  |  |  |  |  |  |  |
| Any other results reported *(e.g. odds ratio, risk difference, CI or P value)* |  | | | | |  |
| Comparison 2 Results | Intervention (____________) | | | Comparison (_____________) | |  |
|  | No. with event | Total in group | | No. with event | Total in group |  |
|  |  |  |  |  |  |  |
| Any other results reported *(e.g. odds ratio, risk difference, CI or P value)* |  | | | | |  |
| No. missing participants |  | | |  | |  |
| Reasons missing |  | | |  | |  |
| No. participants moved from other group |  | | |  | |  |
| Reasons moved |  | | |  | |  |
| Unit of analysis *(by individuals, cluster/groups or body parts)* |  | | | | |  |
| Statistical methods used and appropriateness of these *(e.g. adjustment for correlation)* |  | | | | |  |
| Reanalysis required? *(specify, e.g. correlation adjustment)* | Yes No Unclear | |  | | |  |
| Reanalysis possible? | Yes No Unclear | |  | | |  |
| Reanalysed results |  | | | | |  |
| Notes: | | | | | | |

***Outcome 6*** (pathological complete response)

|  | Description as stated in report/paper | | | | | Location in text or source *(pg & ¶/fig/table/other)* |
| --- | --- | --- | --- | --- | --- | --- |
| Comparison |  | | | | |  |
| Outcome |  | | | | |  |
| Subgroup |  | | | | |  |
| Time point *(specify from start or end of intervention)* |  | | | | |  |
| Comparison 1 Results | Intervention (____________) | | | Comparison (_____________) | |  |
|  | No. with event | Total in group | | No. with event | Total in group |  |
|  |  |  |  |  |  |  |
| Any other results reported *(e.g. odds ratio, risk difference, CI or P value)* |  | | | | |  |
| Comparison 2 Results | Intervention (____________) | | | Comparison (_____________) | |  |
|  | No. with event | Total in group | | No. with event | Total in group |  |
|  |  |  |  |  |  |  |
| Any other results reported *(e.g. odds ratio, risk difference, CI or P value)* |  | | | | |  |
| No. missing participants |  | | |  | |  |
| Reasons missing |  | | |  | |  |
| No. participants moved from other group |  | | |  | |  |
| Reasons moved |  | | |  | |  |
| Unit of analysis *(by individuals, cluster/groups or body parts)* |  | | | | |  |
| Statistical methods used and appropriateness of these *(e.g. adjustment for correlation)* |  | | | | |  |
| Reanalysis required? *(specify, e.g. correlation adjustment)* | Yes No Unclear | |  | | |  |
| Reanalysis possible? | Yes No Unclear | |  | | |  |
| Reanalysed results |  | | | | |  |
| Notes: | | | | | | |

Outcome 7 Clinical response

|  | Description as stated in report/paper | | | | | Location in text or source *(pg & ¶/fig/table/other)* |
| --- | --- | --- | --- | --- | --- | --- |
| Comparison |  | | | | |  |
| Outcome |  | | | | |  |
| Subgroup |  | | | | |  |
| Time point *(specify from start or end of intervention)* |  | | | | |  |
| Comparison 1 Results | Intervention (____________) | | | Comparison (_____________) | |  |
|  | No. with event | Total in group | | No. with event | Total in group |  |
|  |  |  |  |  |  |  |
| Any other results reported *(e.g. odds ratio, risk difference, CI or P value)* |  | | | | |  |
| Comparison 2 Results | Intervention (____________) | | | Comparison (_____________) | |  |
|  | No. with event | Total in group | | No. with event | Total in group |  |
|  |  |  |  |  |  |  |
| Any other results reported *(e.g. odds ratio, risk difference, CI or P value)* |  | | | | |  |
| No. missing participants |  | | |  | |  |
| Reasons missing |  | | |  | |  |
| No. participants moved from other group |  | | |  | |  |
| Reasons moved |  | | |  | |  |
| Unit of analysis *(by individuals, cluster/groups or body parts)* |  | | | | |  |
| Statistical methods used and appropriateness of these *(e.g. adjustment for correlation)* |  | | | | |  |
| Reanalysis required? *(specify, e.g. correlation adjustment)* | Yes No Unclear | |  | | |  |
| Reanalysis possible? | Yes No Unclear | |  | | |  |
| Reanalysed results |  | | | | |  |
| Notes: | | | | | | |

***Outcome 8*** (treatment toxicity)

|  | Description as stated in report/paper | | | | | | | Location in text or source *(pg & ¶/fig/table/other)* |
| --- | --- | --- | --- | --- | --- | --- | --- | --- |
| Comparison |  | | | | | | |  |
| Outcome |  | | | | | | |  |
| Subgroup |  | | | | | | |  |
| Time point *(specify*  *from start or end of intervention)* |  | | | | | | |  |
| Results | Intervention (____________) | | | Comparison (_____________) | | | |  |
|  | No. with toxicity | Total in group | | No. with toxicity | | Total in group | |  |
|  |  |  |  |  |  |  |  |  |
| Type of treatment toxicity | List | Intervention  No of toxicity | | Comparison 1  No. of toxicity | | Comparison 2  No of toxicity | |  |
|  | Serious Infection |  | |  | |  | |  |
|  | Cardio toxicity |  | |  | |  | |  |
|  | postoperative complications |  | |  | | |  |  |
|  | nausea and vomiting |  | |  | | |  |  |
|  | Fatigue |  | |  | | |  |  |
|  | Neurotoxicity |  | |  | | |  |  |
|  | Febrile nutropenia |  | |  | | |  |  |
|  | Other toxicity 1 |  | |  | | |  |  |
|  | Other toxicity 2 |  | |  | | |  |  |
|  | Other toxicity 3 |  | |  | | |  |  |
| Any other results reported *(e.g. odds ratio, risk difference, CI or P value)* |  | | | | | | |  |
| No. missing participants |  | | | |  | | |  |
| Reasons missing |  | | | |  | | |  |
| No. participants moved from other group |  | | | |  | | |  |
| Reasons moved |  | | | |  | | |  |
| Unit of analysis *(by individuals, cluster/groups or body parts)* |  | | | | | | |  |
| Statistical methods used and appropriateness of these *(e.g. adjustment for correlation)* |  | | | | | | |  |
| Reanalysis required? *(specify, e.g. correlation adjustment)* | Yes No Unclear | |  | | | | |  |
| Reanalysis possible? | Yes No Unclear | |  | | | | |  |
| Reanalysed results |  | | | | | | |  |
| Notes: | | | | | | | | |

Other Outcome if reported (Outcome Name:_________________________________)

|  | Description as stated in report/paper | | | | | Location in text or source *(pg & ¶/fig/table/other)* |
| --- | --- | --- | --- | --- | --- | --- |
| Comparison |  | | | | |  |
| Outcome |  | | | | |  |
| Subgroup |  | | | | |  |
| Time point *(specify from start or end of intervention)* |  | | | | |  |
| Results | Intervention | | | Comparison | |  |
|  | No. with event | Total in group | | No. with event | Total in group |  |
|  |  |  |  |  |  |  |
| Any other results reported *(e.g. odds ratio, risk difference, CI or P value)* |  | | | | |  |
| No. missing participants |  | | |  | |  |
| Reasons missing |  | | |  | |  |
| No. participants moved from other group |  | | |  | |  |
| Reasons moved |  | | |  | |  |
| Unit of analysis *(by individuals, cluster/groups or body parts)* |  | | | | |  |
| Statistical methods used and appropriateness of these *(e.g. adjustment for correlation)* |  | | | | |  |
| Reanalysis required? *(specify, e.g. correlation adjustment)* | Yes No Unclear | |  | | |  |
| Reanalysis possible? | Yes No Unclear | |  | | |  |
| Reanalysed results |  | | | | |  |
| Notes: | | | | | | |

# Other information

|  | **Description as stated in report/paper** | **Location in text or source** *(pg & ¶/fig/table/other)* |
| --- | --- | --- |
| **Key conclusions of study authors** |  |  |
| **References to other relevant studies** |  |  |
| **Correspondence required for further study information** *(from whom, what and when)* |  | |
| **Notes:** | | |
